# Variation in avian egg shape and nest structure is explained by climatic conditions

**DOI:** 10.1038/s41598-018-22436-0

**Published:** 2018-03-07

**Authors:** Daisy Englert Duursma, Rachael V. Gallagher, J. Jordan Price, Simon C. Griffith

**Affiliations:** 1 Department of Biological Sciences, Macquarie University, North Ryde, NSW Australia; 2 Department of Biology, St Mary’s College of Maryland, St Mary’s City, MD USA

## Abstract

Why are avian eggs ovoid, while the eggs of most other vertebrates are symmetrical? The interaction between an egg and its environment likely drives selection that will shape eggs across evolutionary time. For example, eggs incubated in hot, arid regions face acute exposure to harsh climatic conditions relative to those in temperate zones, and this exposure will differ across nest types, with eggs in open nests being more exposed to direct solar radiation than those in enclosed nests. We examined the idea that the geographical distribution of both egg shapes and nest types should reflect selective pressures of key environmental parameters, such as ambient temperature and the drying capacity of air. We took a comparative approach, using 310 passerine species from Australia, many of which are found in some of the most extreme climates on earth. We found that, across the continent, egg elongation decreases and the proportion of species with domed nests with roofs increases in hotter and drier areas with sparse plant canopies. Eggs are most spherical in open nests in the hottest environments, and most elongate in domed nests in wetter, shadier environments. Our findings suggest that climatic conditions played a key role in the evolution of passerine egg shape.

## Introduction

The classic ovoid egg shape of birds is one of the most familiar of all shapes, and yet it remains surprisingly poorly understood^1,2^. The eggs of most other oviparous vertebrates are much more symmetrical and often more spherical^2,3^. The extent of our ignorance about the adaptive significance of egg shape in birds was recently highlighted when the long-standing myth that the eggs of Common Guillemots (*Uria aalge*) are pear-shaped in order to prevent them from rolling off cliff ledges was corrected^4^. Although this was an intuitively appealing idea, in fact the guillemot’s egg is not strikingly different in shape from the eggs of most other birds, and relatively few birds lay their eggs on cliff ledges, suggesting that some more widely applicable explanations remain to be found for the ubiquitous ovoid shape of birds’ eggs^1,4^.

A recent comparative study of 1400 bird species identified a link between egg shape and the flight abilities of different species^2^. Egg shape can also vary among species due to the co-evolution of avian brood parasites and their hosts^5^. Although these analyses considered a range of biophysical, life history and ecological parameters, previous comparative studies have not considered the abiotic environment into which an egg is laid, which includes a wide range of factors that may be detrimental to the development of an embryo. For instance, eggs must remain within a relatively narrow temperature range^6^, and a balance must be found between gaseous exchange and dehydration^7^. Many avian species breed successfully under extreme climatic conditions, including hot and arid environments where extreme temperatures augment the drying capacity of the air. In addition, the paucity of vegetation in such habitats reduces the amount of shade available, increasing the potential for intense solar radiation to affect the egg and the embryo developing within.

The design of an egg should be optimised for the environmental conditions to which it is exposed^8^. For example, birds breeding in deserts, at high altitudes, or at high temperatures have a relatively lower shell conductance, limiting water loss whilst also permitting gaseous exchange^9,10^. But there is a lower limit on eggshell conductance and permeability, given the need for effective exchange of oxygen and carbon dioxide between the embryo and the atmosphere. For any given volume, a spherical egg has a lower surface area to volume ratio than does an ovoid egg^11^. Therefore, if all else is equal, a spherical egg will gain and lose heat more slowly, lose less water, and have lower exposure to solar radiation than will a more elongate egg with the same volume. In brood parasites it has been demonstrated that the small surface-area-to-volume ratio of spherical eggs is optimal for heat retention, increasing the speed of development relative to those of their hosts^12–14^. In the well-studied domestic chicken (*Gallus gallus domesticus*) rounder eggs have thicker shells^15^, but the relationships between elongation and shell conductance has not been well explored at a broader level^8^.

Nest relative humidity and the water vapour pressure difference (${{\rm{P}}}_{{{\rm{H}}}_{{\rm{2}}}{\rm{O}}}$) across the eggshell are known to affect water loss from eggs^16–18^. The size, structure and composition of nests – along with the brood patch of incubating parents – all contribute to the microclimate around an egg, with differences in vapour pressure between egg and nest microclimate largely determining the degree of water loss^19^. Experimental studies have shown that changes in nest relative humidity can reduce egg hatchability^20,21^. An obvious way in which a species can reduce or buffer exposure of the egg to the wider environment is through the design of the nest. Nests that are enclosed (e.g. those in cavities or burrows, or domed structures with roofs) should be beneficial in the maintenance of desirable temperatures and levels of relative humidity in hot and arid regions^22^. Whilst parents can also shape the thermal and humidity profile of their nest through their incubation behaviour, (for example, adjusting the contact area between the incubating parent and the egg, and the time spent in the nest), at high temperatures (31 °C–40 °C^23^), birds may reduce their attendance at the nest as the requirements for heat transfer to the egg are reduced^23,24^. Thus the regulatory effects of enclosed nests on nest microclimate may be of critical importance in hot arid environments.

It has been hypothesised that nests can function as incubators in which optimal conditions for egg development are maintained^19^. Here, we assume that in hot and arid regions enclosed nests have the greatest potential to maintain optimal nest temperatures and levels of relative humidity^25^. This increases the probability that not too much water is lost from the egg and that temperatures are not too high over the course of development. Further, enclosed nests provide shade in areas with little vegetation cover, reducing the likelihood that incubating birds and eggs overheat when exposed to direct sunlight^9^. Indeed, in the arid zone of Australia, experimental removal of the roof of Zebra Finch (*Taeniopygia guttata*) nests results in a significant increase in temperature in the nest cup, and orienting the nest entrance towards or away from direct sunlight also significantly affects internal temperatures^26^.

In the passerines (Passeriformes), nest form is largely phylogenetically conserved across taxa, with transitions between domed nests and open cup nests occurring relatively rarely during passerine evolutionary history^27^. On the Australian continent, the geographic origin of the oscine passerines^28^, 33.7% of passerine species build dome-shaped nests, and phylogenetic analyses indicate that this ancestral form has remained largely unchanged in some lineages for tens of millions of years^27^. Cavity nesting has evolved in relatively few Australian taxa^27^. Phylogenetic relatedness is only one explanation for the current-day patterns in species traits^29^ and given this evolutionary inertia in nest form, it seems likely that specific combinations of nest and egg shapes would be advantageous and help species adapt to different environments, particularly extreme climatic conditions (e.g. very wet, dry or hot). Thus we expect certain combinations to be more common under some environmental conditions (e.g. domed nests and rounder eggs should be more common in hot dry conditions). We acknowledge that variation in incubation behaviour across species might also contribute to the maintenance of an optimal microclimate for eggs in a nest. However, in the absence of accurate data across species over the extent to which the presence of adults affects the microclimate of the nests, and the amount of water and heat lost from unattended eggs, we have assumed that such parental influences are on average equal across climates and nest types. The extent to which this assumption is true will be a useful focus of future work.

Given that gaseous exchange renders an egg vulnerable to water loss, we expect egg shapes that reduce overall surface area to dominate where environmental conditions are hot, dry and sparsely vegetated to minimize water loss and exposure to UV radiation. Furthermore, given that the three distinct nest types (cavities, open cup and domed nests) provide different levels of protection against exposure to climatic conditions, we would expect the relationship between environmental conditions and egg shape to differ with nest structure.

We test these ideas at a continental scale across the assemblage of Australian passerines. Assessing traits, and geographic variation of traits across assemblages of species is a way to assess ecological strategies that optimize fitness under a set of environmental conditions. It has been demonstrated that geographic variation in functional traits can largely be explained by environmental factors^30,31^. Australia provides an ideal opportunity to assess variation in bird traits and phenology because of its wide range of environmental conditions, distinct biogeography and because, relative to other continents, it contains a large proportion of the Passerine families, being the host of the early evolutionary radiation of this important group that constitutes more than half of all avian species. Although a large portion of the country is covered by arid vegetation, there are also tropical, subtropical, and temperate biomes^32,33^.

**Figure 1:**
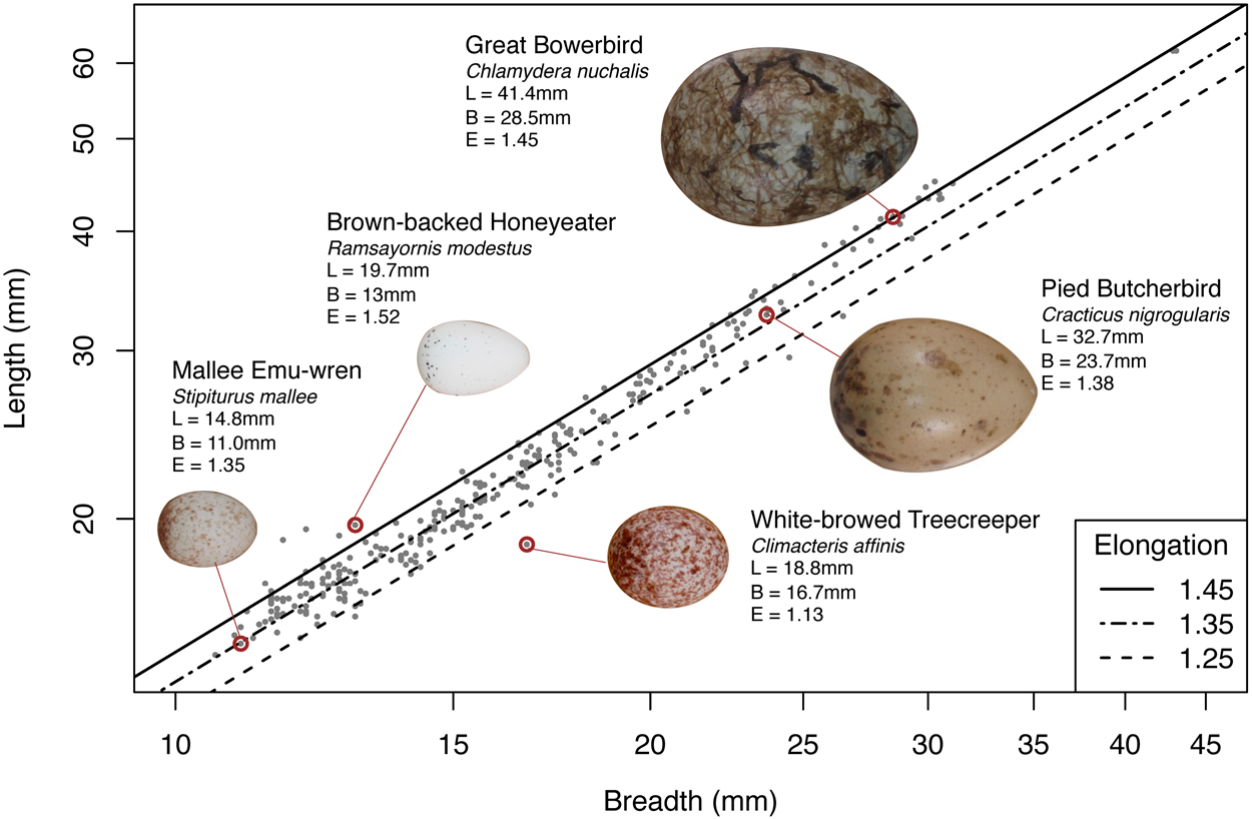
The relationship between egg length (L) and breadth (B) for Australian passerine species. The diagonal lines show egg elongation (E), the ratio of the egg length to breadth) at values of 1.25, 1.35, and 1.45.

This is the first comprehensive examination of how egg shape varies for a range of species (Fig. 1) in an explicit spatial context. We combine records of avian breeding locations (e.g. ATLAS records and nest record schemes) with data from museum egg-collections, bird-banding records, and citizen science initiatives^34^ to determine the geographic extent of breeding for 310 species of passerine birds occurring in 100 km × 100 km grid cells across Australia. This information is coupled to data on two key functional traits: egg elongation (the ratio of the egg length to breadth) and nest type (cup-shaped, dome-shape with roof, cavity). We examined how avian nest type and egg shape vary across the Australian continent in relation to two environmental variables important for understanding climatic stress: average vapour pressure deficit (VPD) and leaf area index (LAI). In particular, we focused on understanding strategies for successful breeding in hot and arid environments. In the arid zone of inland Australia, for example, where tree cover is typically sparse, we expect natural selection to have favoured traits beneficial for reducing water loss, maintaining eggs below critical upper temperature limits, and decreasing exposure of the incubating birds and embryos to solar radiation.

## Material and Methods

### Geographic Distributions

The geographic breeding ranges for 310 native Australian mainland passerine species were compiled using occurrence observations (latitude and longitude coordinates) from Birdlife Australia’s Atlas^35^ and Nest Record Scheme, historical museum egg collection records, Australian Bird and Bat Banding Scheme, Atlas of Living Australia^36^, Global Diversity Information Facility^37^, and eBird^38^. Using the breeding observations we determined the geographic distribution of each species at a grid cell resolution of 100 km × 100 km in Albers Equal Area Conic Projection. The geographic distributions of all species were used to determine species assemblages for each 100 km × 100 km grid cell. A species assemblage is all the species known to breed in each 100 km × 100 km location. The geographic distributions are available from Supplementary Table S1 via 10.6084/m9.figshare.5579398. For a complete list of contributing institutes and persons, detailed methodology of how data were cleaned, and geographic distributions were determined, please see Supplementary Material.

### Trait Data

We gathered information on egg elongation – the ratio of egg length to breadth – from three sources: (1) published values in the Australian Bird Data Version 1^39^, (2) by directly measuring eggs at the Australian National Wildlife Collection (Canberra, Australia: http://www.csiro.au), and (3) using photos of eggs from the Online Zoological Collections of Australian Museums (http://ozcam.ala.org.au). Egg elongation was calculated for 308 of the species; the remaining two species, Green-backed Honeyeater (*Glycichaera fallax*) and Kalkadoon Grasswren (*Amytornis ballarae*), were dropped from the study because we lacked information on egg length and breadth.

We classified nest type for species as either cup-shaped, domed, or cavity using published sources^27^. Cup-shaped nests were defined as those with the upper portion exposed (*n* = 190), while domed nests have the upper proportion enclosed with a constructed roof, including globular and pendulous nests with side entrances^40,41^ (*n* = 102). Cavity nesters (*n* = 16) include those nesting in burrows or hollows in locations such as tree holes, rock cavities, or holes in the ground. The majority of cavity nesters occur in the families Climacteridae, and Pardalotidae. Two species made highly variable nests (*Mirafra javanica* and *Cisticola juncidis*) that are typically partly or fully roofed^42,43^. We classified these species as domed. See Supplementary Table S2 via 10.6084/m9.figshare.5579398 for species level information.

To assess the spatial pattern of variation of average egg elongation across Australia, we found the mean elongation for species with cup-shaped nests and species with domed nests for each 100 km × 100 km grid cell species assemblages (i.e. the species breeding in each 100 km × 100 km grid cell). Cavity nesting species had significantly rounder eggs than species with domed and cup-shaped nest, and given there were only 16 species, we excluded them from the grid cell species assemblage’s analyses. For each grid cell we also found the proportion of the species assemblages with domed nests.

### Environmental Data

To assess the relationships between environmental conditions and functional traits, we calculated average daily maximum vapour pressure deficit (VPD) and average Leaf Area Index (LAI) for the Australian continent, at a 100 km × 100 km grid cell resolution.

**Figure 2:**
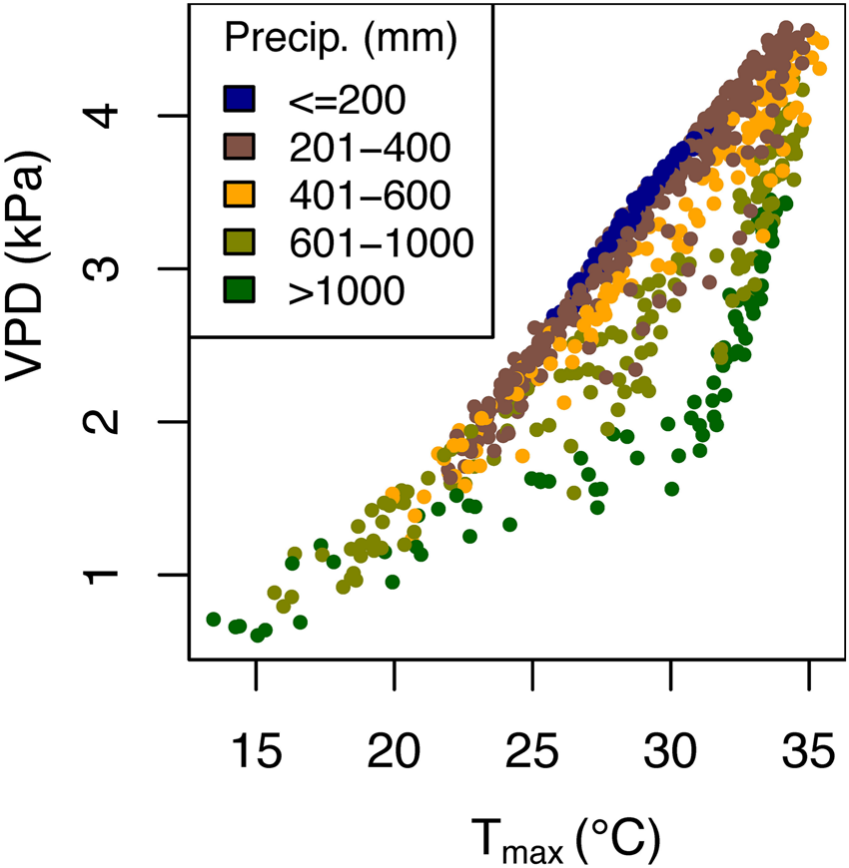
Relationship between mean ambient air temperature (T_max_, °C), mean vapour pressure deficit (VPD, kPa), and mean monthly total precipitation (Precip) for the Australian continent at 100 km × 100 km grid cell resolution. Data were downloaded from Australian Water Availability Project^44^ via http://www.bom.gov.au/jsp/awap/ and averaged across the time period 1950 to 2016.

**Figure 3:**
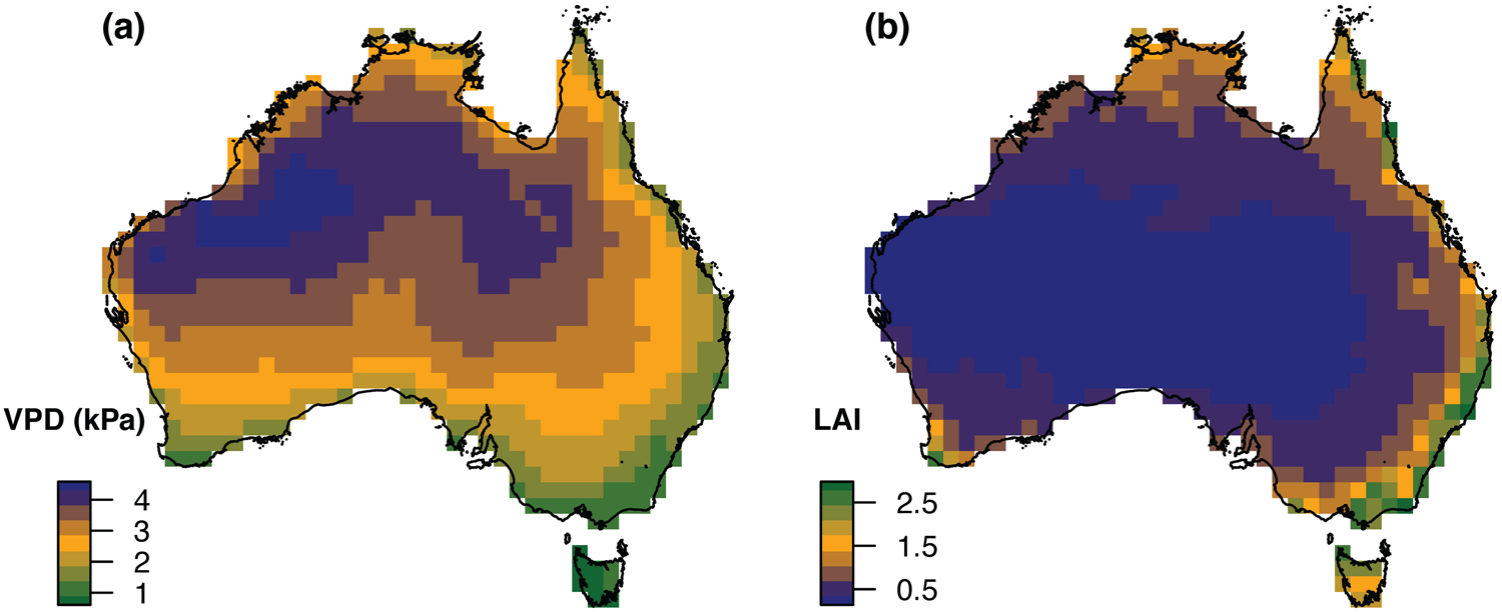
Variation in (**a**) average annual vapour pressure deficit (VPD, kPa) and (**b**) average annual leaf area index (LAI) for the Australian continent. Maps show average values for 100 km × 100 km grid cells. Maps were created in R 3.3.2^47^ using raster^72^ and base plotting functions. Digital boundaries of Australia are from Australian Standard Geographical Classification (ASGC) (cat. no. 1216.0) via http://www.abs.gov.au/.

We defined VPD as the difference between the saturated vapour pressure at daily maximum temperature (T_max_) and daily vapour pressure (VP) at 3 pm. T_max_ and VP were downloaded from Australian Water Availability Project^44^ via http://www.bom.gov.au/jsp/awap/. The *esat* function in the R library *plantecophys*^45^ was used to convert daily maximum temperature to saturated vapour pressure. Data were averaged across the time period 1950 to 2016, projected to Albers Equal Area Conic Projection, and aggregated to 100 km × 100 km grid cells. VPD is potentially of critical importance in the maintenance of nest humidity and eggshell conductance. VPD is a function of the amount of water vapour in the air and ambient temperature. As temperatures increase and VP remains constant, the VPD, or the air’s ability to dry, increases (Fig. 2). The Australian continent has a large range of average annual VPD (Fig. 3a), with moist temperate regions having values close to 0 kPa, and arid, warmer regions having values over 4 kPa.

LAI is the amount of green leaf area in relation to ground area^46^. Areas with higher values of LAI, such as the tropical region of Australia (Fig. 3b), have greater canopy cover protecting nests and eggs from UV radiation throughout the day. In open environments, with lower values of LAI, more direct sunlight reaches the ground, and nests are likely to be exposed to some direct solar radiation, although nest position can influence the amount of UV radiation a nest is exposed to, and even in very open environments a nest can be well shaded. LAI values used in this study were satellite-derived and indicated the number of leaf layers per unit ground. LAI measurements for 16-day intervals during the period February 2000 to 2016 were obtained via the TERN AusCover portal (http://www.auscover.org.au) and were produced from tiles originally downloaded from USGS (https://lpdaac.usgs.gov). Data were averaged across the time period and aggregated to 100 km × 100 km resolution following the process above.

### Analyses

Three specific statistical analyses were performed to examine: (1) the relationship between egg elongation and nest type across species when geographic distribution was not considered (2) spatial variation in grid cell averages of egg elongation for species assemblages in relation to VPD and LAI, and (3) spatial variation in the proportion of species in the species assemblages with domed nests in relation to VPD and LAI. All analyses and figures were carried out in R 3.3.2^47^ and relationships were considered significant at an alpha level of 0.05. Throughout, summary values are reported as means ± SE.

ANOVA was used to test for differences in elongation among nest-types both at the species level and for the 100 km × 100 km species assemblages, with Type II Wald *F* tests and Kenward-Roger approximation for degrees-of-freedom using the *car* package^48^ and Tukey’s pairwise comparisons using the *multcomp* package^49^. To test for bias in the species level model from shared ancestry, we also performed the species level analysis using a linear mixed-effects model, using *lme4* package^50^, with nest-type as fixed effects and taxonomic family as a random effect. We found no meaningful difference in the results (see Supplementary material), thus we only report the results from the ordinary least squares (OLS) regression.

We assessed the variation in (i) proportion of species with domed nests and (ii) mean egg elongation for grid cell assembles of species in domed nests and species in cup-shaped nest, in relation to VPD and LAI. We used OLS regression and a simultaneous autoregressive model (SAR). In all regression models, we included an interaction between LAI and VPD.

Spatial autocorrelation of model residuals violates a key assumption of statistical analysis, i.e., independence of residuals and identical distribution^51,52^. Moran’s *I* statistic, in the *ncf* package^53^, was used to detect spatial autocorrelation in the model residuals (i.e., values with close spatial distribution are more similar than expected when compared to those that are of further distance apart). Where spatial autocorrelation was detected, we used a simultaneous autoregressive model (SAR) to add an autocorrected error term of spatial weights using the package *spdep*^54,55^. A row standardized spatial weight matrix was calculated using a neighborhood distance of 200 km. These parameters were selected because they returned low values of Moran’s *I* statistic over the first 20 distance groups when coding schemes of binary and row standardised were tested, in preliminary analysis to calculate spatial weights using neighbourhood distances of 200 km, 300 km, 400 km, 500 km, 750 km, and 1000 km.

### Data availability

The data used in this manuscript are publically available (i.e., climatic data and occurrence observations). Observations were collated from a variety of online databases, institutes, and persons (for a complete list institutes and persons, please see the Supplementary Material). Although most of this data are freely available, redistribution is restricted by license. To make this study reproducible and to adhere to licensing agreements, we have made available gridded occurrence of breeding passerines in Australia at a 100 km × 100 km resolution (Supplementary Table S1) and trait data (Supplementary Table S2). Data is available from: 10.6084/m9.figshare.5579398.

## Results

Across the Australian passerine species, egg shape varies markedly, from nearly spherical (1.13 for the White-browed Treecreeper (*Climacteris affinis*)) to highly elongated (1.62, much greater length than breadth, for the Dusky Gerygone (*Gerygone tenebrosa*)), with an overall mean elongation of 1.37 ± 0.004 (Fig. 1, species level data available in Table S2). Egg elongation for the 308 species and for within grid cell species assemblages (i.e. the species breeding in each 100 km × 100 km grid cell) were normally distributed, although three species that had very elongated eggs fell outside of the normal distribution. Therefore, we performed separate analyses for: (i) all species, and (ii) species with egg elongation values within three standard deviations of the mean. We found no meaningful difference between the results for these two analyses, so we only report results across all species.

In the assessment of nest type and egg elongation, when the geographic distribution of species was not considered, there was significant variation in egg elongation across all species ([F (2, 305) = 10.59, p < 0.001]). Cavity-nesting species had significantly less elongated eggs (p < 0.001, 1.30 ± 0.02, *n* = 16) than species that nest in cup-shaped and domed nests (1.37 ± 0.02, *n* = 191; 1.37 ± 0.02, *n* = 101, respectively); however, there was no significant difference in elongation between cup-shaped and domed nesting species (p = 0.95). See Table S2 for species level data including nest type.

**Figure 4:**
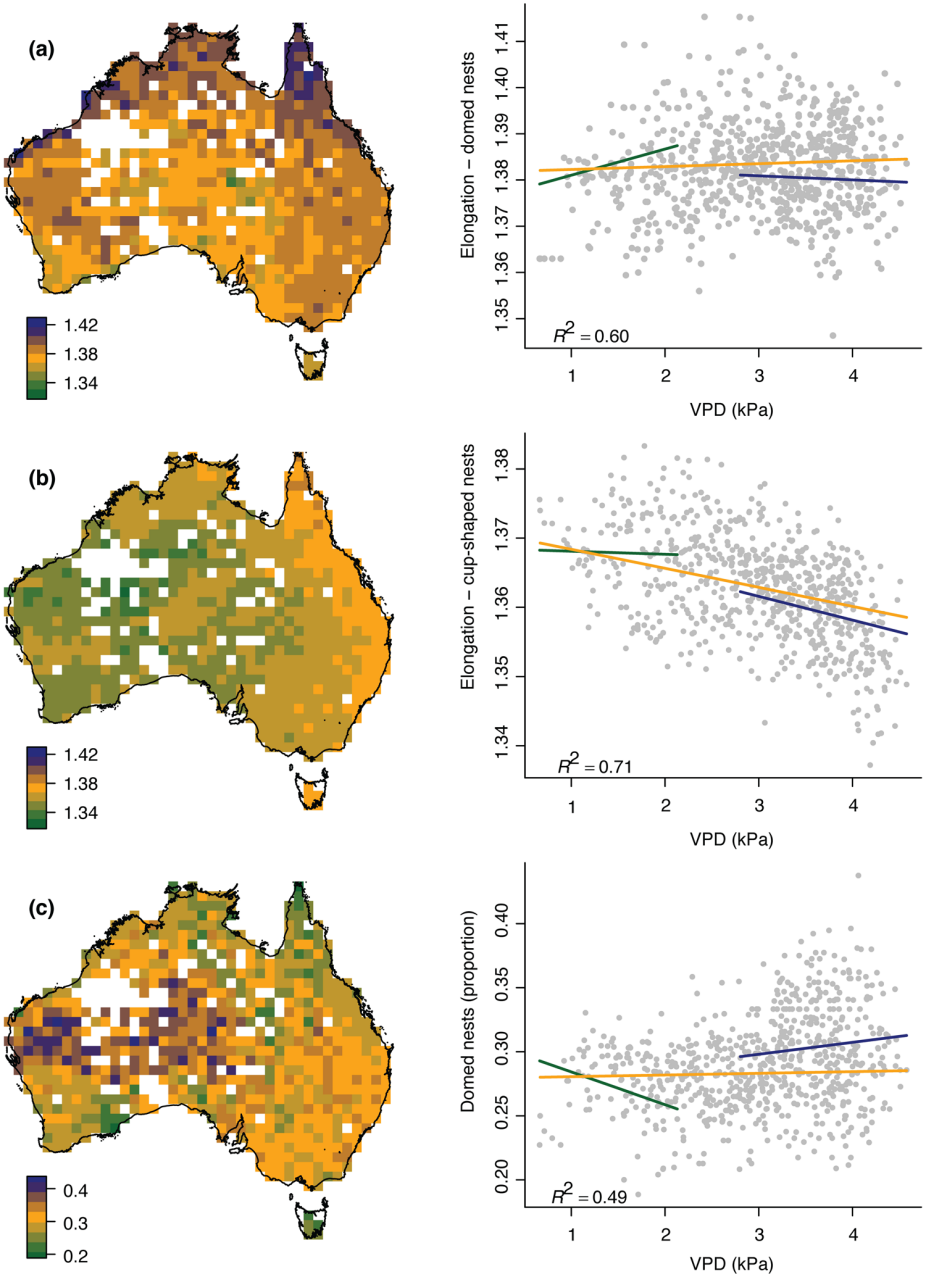
Variation in egg elongation and the proportion of species with domed nests for Australia’s passerines. The maps depict the spatial variation in 1° × 1° grid cells of (**a**) mean egg elongation for species with domed nests, (**b**) mean egg elongation for species with cup-shaped nests, and (**c**) the proportion of species with domed nests. Maps were created in R 3.3.2^47^ using raster^68^ and base plotting functions. Digital boundaries of Australia are from Australian Standard Geographical Classification (ASGC) (cat. no. 1216.0) via http://www.abs.gov.au/. White grid cells either do not meet the required Completeness Index (i.e. the ratio of the observed species richness to the estimated species richness, see Supplementary Material), or had less than ten species with domed nests or less than ten species with cup-shaped nests. Plots show the relationship between mean grid cell values (response variables) and vapour pressure deficit (kPa). Colored lines depict the predicted response from the simultaneous autoregressive models when LAI is at the mean (yellow line), below the 5^th^ percentile (blue) above the 95^th^ percentile (green line). Nagelkerkes pseudo R^2^s are given.

When the geographic distribution of species was considered, we found that for assemblages of species that occur in 100 km × 100 km gridcells, avian eggs were less elongated and domed nests were more common in areas that are hot and dry (high VPD) and have sparse plant canopies (low LAI). Mean egg elongation for grid cell assemblages of species with domed nests ranged from 1.35 to 1.42 (mean: 1.38 ± 0.009, Fig. 4a) and for cup-shaped nests ranged from 1.33 to 1.38 (mean: 1.36 ± 0.008, Fig. 4b). Egg elongation for assemblages of species with domed nests and species with cupped nests were significantly different ([F (1, 711) = 90.2, p < 0.001]), with those in cupped nests generally being rounder at any location (Fig. 4a,b). Mean elongation for species with domed nests was greatest for the tropical biome of Australia (based on a national modified Köppen classification system, Stern *et al*.^32^, BOM 2006), with values greater than the 80% quantile (1.390) predominately located there. Species with cup-nests had a different pattern, with mean elongation values above the 80% quantile (1.368) located across eastern Australia. For both nest types, the grid cells with the lowest values of mean elongation were predominately located in the desert and grassland biomes. Likewise, the proportion of species with domed nests was greatest in the hot and dry areas, with nearly all grid cells above the 80% quantile (0.317) located in the desert (Fig. 4c). The proportion of species with domed nests varied from 0.19 to 0.44 (mean: 0.29 ± 0.035; Fig. 4c).

LAI and VPD explained a significant amount of the variation in mean egg elongation for species in domed nests (r2 = 0.71), mean elongation for species in cupped nests (r2 = 0.60), and in the proportion of species with domed nests (r2 = 0.49, [for variable significance see Table 1]). Based on the SAR models of these variables, as VPD increased and LAI decreased: (i) mean egg elongation for species in cup-shaped and in domed nests both declined (i.e. eggs became significantly rounder), and (ii) the proportion of species with domed nests increased (Fig. 4). However, for species with domed nests, mean elongation increased under conditions with moderate VPD and high LAI. These conditions can occur in the tropics.

**Table 1:** The importance of VPD, LAI, and their interaction for explaining variation in mean elongation for species with domed nests and for those with cup-shaped nests, and the proportion of species with domed nests. Elongation is the ratio of egg length to egg breadth. *Indicates a variable that contributed significantly to model (p < 0.05).

Model	Variable	Mean (±SE) SAR model coefficients
Elongation – Species with domed nests	VPD	1.382 ± 0.001
	LAI*	1.378 ± 0.002
	VPD:LAI*	1.388 ± 0.002
Elongation – Species with cup-shaped nests	VPD*	1.369 ± 0.001
	LAI	1.370 ± 0.001
	VPD:LAI	1.374 ± 0.001
Proportion of species with domed nests	VPD*	0.279 ± 0.004
	LAI*	0.288 ± 0.009
	VPD:LAI*	0.245 ± 0.006

## Discussion

To the best of our knowledge this is the first continental study of functional avian traits. We found that in the hot, dry, and sparsely vegetated interior of Australia (high VPD, low LAI): (i) the proportion of species with domed nests was greater (Fig. 4c) and (ii) average egg elongation was lower than that found across the rest of Australia (Fig. 4a and b). This variation in egg elongation and nest type in response to VPD and LAI indicates how, at the landscape level, strategies for reducing stress in hot and dry environments are favoured in bird assemblages.

We identify an association across bird assemblages between high abiotic stress (in deserts) and the occurrence of rounder eggs, particularly in open nests. The potential advantages of round eggs under hot and dry climatic conditions have not been well explored, and elongation and asymmetry has often been related to nest type, space occupied in clutch, and ability to roll^11,56^. Based on current research we hypothesise three potential benefits to rounder eggs in desert regions. First, it is likely that the shape of eggs is acting as a surrogate for traits that affect shell conductance. Shell conductance and pore density vary across an egg, with the blunt end of eggs having greater values^57^. It has been found that pore size affects the incubation rates of birds in temperate regions^58^, although the evidence is mixed^59,60^ and may not be relevant to desert regions. Future research is needed to investigate whether there is a relationship between egg elongation, which is a trait readily available for many species, and shell conductance. It is worth noting that intraspecific variation in egg shape is linked to shell thickness in poultry eggs, with rounder eggs having thicker shells. Thick eggshells have greater pore length and, according to Fick’s law of diffusion, this decreases shell conductance. Second, we hypothesise that the heat capacity of elongated eggs is less than that of round eggs^6^; therefore, elongated eggs warm and cool more quickly than round eggs. Third, round eggs in cup-shaped nests in open environments have a lower proportion of surface area receiving radiation to total surface area ratio compared to eggs that are highly elongated under the same conditions, as shown for different animal shapes^61^. This reduces heat transfer from thermal radiation and exposure of the embryo to potentially harmful UV radiation^62^.

The methods and results presented here complement laboratory-based experiments on critical temperature thresholds and variable humidity for eggs and embryos. These experiments have been limited to looking at embryonic stress for a small number of species in field or laboratory-based settings^21,63^. However, understanding variation in traits at large-scales, across many species, is important for practical activities such as estimating the potential impact of climate change^31,64,65^. Identifying traits that may be advantageous when species are breeding in extreme conditions, such as high temperatures with little precipitation, is crucial for providing a more holistic understanding of species responses to climatic drivers.

The spatial variation in nest type that we see across Australia supports the idea that nests act as incubators where temperature, relative humidity, and respiratory gasses are maintained within optimal conditions^19^. In hot, dry, and open environments, domed nests: provide shade, benefiting both the incubating bird and embryos through reduced temperatures^26^; reduce the vapour pressure difference between the ambient air and the eggs^25^; and provide shelter from the wind (reviewed in^25^). The connection between nest type and the regulation of water loss and temperature of eggs has been well established^19,25,66^. Recently it has been proposed that multiple selective drivers have acted upon nest architecture and that there are two main functional properties of nests^19^. The first function is for the conservation of heat and the second is to maintain the appropriate microclimate^19^. In the desert and grassland regions of Australia, it seems likely that the primary function of nests is for the maintenance of suitable microclimates, particularly higher relative humidity and lower temperatures. Given the lack of change in nest structure over long periods of evolutionary time^27^, we propose that dome-building species are better adapted to living in harsh arid environments and are therefore more likely to immigrate into those regions, rather than the alternative explanation that species modify their nest structures during or following immigration.

Our findings from hot and arid desert regions contrast markedly to those from tropical regions within Australia. Tropical regions typically have very high LAI, i.e. large areas of closed canopy forest. During the rainy season, VPD can be close to 0 kPa while during the dry season VPD can be quite high with average annual VPD around 2.5 kPa^44^. Species that nest in very wet conditions (i.e. grebes, Gaviiformes) have many pores in their eggshells to allow for adequate water loss and gas exchange^18^. In areas with seasonally low levels of VPD, e.g. wet conditions, fewer passerine species make domed nests (~25% of species, Fig. 4c), especially when compared explicitly to arid regions (~40% of species). This is likely because domed nests may absorb excessive water, resulting in the nest relative humidity being high and adequate gas exchange inhibited, as shown in studies where nests were exposed to air saturated with moisture^20,21^.

The need to meet adequate gas exchange may also explain why birds with domed nests in the tropics have, on average, the highest values for elongation (above the 80% quantile, Fig. 4a), which may facilitate gas exchange. The amount of water an egg loses during incubation is a combination of the water vapour pressure difference between the egg and its microclimate, egg size, incubation duration, and shell conductance^10,21,25,67,68^. Whilst birds do not actively control for water loss from eggs, they can influence water loss through nest site selection, nest type, nest material, nesting behaviour and the timing of breeding^17,56,69,70^. The appropriate loss of water from avian eggs during incubation is directly related to the successful development of embryos^56,71^, and for most species water accounts for 15% of the initial egg mass^10^. We were unable to control in any way for parental behaviour during the egg stage, and acknowledge that this remains a good target for future research. It is certainly possible that parental incubation or egg caring behaviour may vary persistently across species with respect to both the environment in which they live, and the type of nest that they build. Nonetheless, our findings suggest that even if they do vary in such a persistent way, their behaviour is unable to completely compensate for the different environmental selective pressures that occur across these axes of variation.

Our study suggests that nest type and egg elongation vary under large-scale changes in VPD and LAI, particularly under extreme values. This variation in nest type and egg elongation may provide insight as to which species may be better suited to overcome the potential challenges faced under extreme weather events, particularly heatwaves. This area of research would benefit from experimental studies aimed at investigating intra-specific variation in nest-shape and egg elongation to further explore whether there are relationships within species among these factors, gas exchange rates, and embryonic temperatures.

## Electronic supplementary material

Supplementary Material
