# Supplementary material for: Variation in avian egg shape and nest structure is explained by climatic conditions
Source: Sci Rep. 2018 Mar 7;8:4141. doi: 10.1038/s41598-018-22436-0 (PMC5841347; doi:10.1038/s41598-018-22436-0)
Supplement: Supplementary file 1 — Supplementary Material [file 41598_2018_22436_MOESM1_ESM.doc]

**Supplementary Information for the manuscript:**

**Variation in avian egg shape and nest structure is explained by climatic conditions**

Daisy Englert Duursma^*a^, Rachael V. Gallagher^a^, J. Jordan Price^b^, Simon C. Griffith^a^

^a^ Department of Biological Sciences, Macquarie University, North Ryde, NSW, Australia

^b^ Department of Biology, St Mary’s College of Maryland, St Mary’s City, MD, USA

^*^*Correspondence author:* daisy.duursma@gmail.com

**Institutes and persons that contributed data**

Australian breeding bird occurrence records were collated from Atlas of Living Australia, Australia Bird and Bat Banding Scheme (ABBBS), Australian Museum, Australian National Wildlife Collection, BirdLife Australia’s ATLAS and Nest Record Schemes, eBird, Global Biodiversity Information Facility, Museum and Art Gallery of the Northern Territory, Museum Victoria, Online Zoological Collections of Australian Museums, Queen Victoria Museum and Art Gallery, Queensland Museum, South Australian Museum, Tasmanian Museum and Art Gallery, and Western Australian Museum. Organizations and individual that contributed the ABBBS data are The Antartic Division, The Bird Care And Conservation Society, The Broome Bird Observatory, Gippsland Lake Ornithological Group, The Gluepot Reserve Study Group, The Western Australian Rehabilitation Group, The Wild Bird Rehabilitation Group, R Aitken, SWW Alford, NP Ali, P Allan, TH Alley, G Ambrose, SJ Ambrose, NW Arnold, H Aston, CN Austin, GB Baker, DJ Baker-Gabb, H Bakker, KA Barker, DE Barnes, IM Bateman, H Battam, G Beal, C Beckmann, H Bell, GD Bell, C Bennett, CM Bishop, CA Bissell, EF Boehm, PF Bolger, AR Bougher, BFJ Bowen, GM Bowker, AN Boyle, JB Bradley, HA Bradley, HE Brenton, RJ Broad, L Broadhurst, MG Brooker, RJ Brown, AH Burbidge, TC Burton, CG Burtt, BM Cale, GR Cam, A Cam, AC Cameron, RG Cameron, CB Campion, M Carins, RK Carruthers, BS Carter, NT Carter, BG Chaffey, GS Chapman, FRH Chapman, BJ Chudleigh, GP Clancy, GF Claridge, GS Clark, M Clayton, A Cockburn, SJ Comer, P Congreve, R Cooper, V Cooper, RE Corbould, DM Cornish, JE Courtney, ML Courtney, PP Coventry, RJ Cox, MM Crouther, JM Cullen, HS Curtis, MR Daley, P Dann, VD Davenport, PM Davidson, SJJF Davies, CPS De Rebeira, SJS Debus, J Dell, DGH Dent, PA Disher, HJ Disney, VA Doerr, DF Dorward, DD Dow, WMCK Dowling, HA Doyle, FW Doyle, PO Doyle, R Draffan, JN Dunlop, RJ Edge, NN Ellis, WB Emison, RC Evans, NJ Favaloro, PJ Fell, J Fennell, LWC Filewood, JH Fisher, KA Fisher, MR Fleming, TI Fletcher, L Fontanini, HA Ford, JR Ford, N Forde, JC Fordyce, B Foreman, PJ Foster, MT Fox, D Franklin, C Frith, DW Frith, HJ Frith, JL Gardner, JA Gates, DJ Geering, RM Gibbs, D Gibson, B Glover, AW Goldizen, RC Good, MD Gottsch, CN Gove, RJP Gower, I Grant, MR Grant, RH Green, K Green, D Green, R Gregory-Smith, MO Gunn, RE Gurney, TH Guthrie, AM Gwynn, LC Haines, C Hall, ML Hall, JH Hall, RH Hardie, JL Hardy, JW Hardy, JGK Harris, CF Heathcote, RG Heinsohn, NL Hermes, AL Hertog, PJ Higgins, MP Hines, WB Hitchcock, CY Ho, JN Hobbs, CR Hodge, DM Hodges, AJ Hole, C Hollamby, JB Hood, I Hore-Lacy, GM Horey, W Horton, ES Hoskin, K Hough, TH Hughes, BR Hutchins, J Hyett, JC Ipsen, J Izzard, A J Leishman, P Jackson, BR Jahnke, A Jansen, PR Johnson, FE Jones, DN Jones, R Jordan, JO Karubian, K Keith, JW Kellam, BJ Kentish, RF Kenyon, J Kikkawa, J Klapste, WL Klau Oam, SM Kleindorfer, SG Lane, NE Langmore, AFC Lashmar, G Laybourne-Smith, S Legge, N Lenz, JE Lewis, AJ Ley, J Liddy, A Lill, E Lindgren, LC Llewellyn, TG Loffler, GJ Logan, NW Longmore, RG Lonnon, KW Lowe, HF Macarthur, D Macdonald, KJ Mack, DB Mack, DB Mack, WDF Mackenzie, RG Mackenzie, MJ Magrath, RD Magrath, WJ Maher, PN Maher, RE Major, EB Male, S Marchant, AJ Marsland, G Marston, RJ Martin, JS Martyn, WE Matheson, KL Maute, I Mccallum, PG Mcdonald, PDD Mcintosh, JL Mckean, JA Mcnamara, MV Melvin, PW Menkhorst, CW Meredith, ALA Middleton, WGD Middleton, PJ Milburn, GJ Millard, DR Milledge, CDT Minton, GJ Moors, B Morgan, AK Morris, OPP Mueller, RA Mulder, MT Murn, SA Murphy, D Murray, MD Murray, JL Nicholls, HJ Nicholson, LA Nielson, T Norostrom, AY Norris, L O'Connor, MP Park, GR Park, CJ Parmenter, VJ Pattemore, JD Patterson, DL Pepper-Edwards, TW Pescott, D Peters, AM Peters, M Pickett, FG Pinchen, D Pinner, T Poldmaa, JM Poole, DJA Portelli, KJ Pound, EK Pratt, JG Pridham, SG Pruett-Jones, SR Pryke, D Putland, GH Pyke, MK Rathburn, J Rawlins, HF Recher, AJ Reid, R Reid, DR Reid, PN Reilly, JW Reside, C Rich, DC Richards, DJ Ripper, D Robinson, VA Robinson, JL Rogan, JH Rooke, HM Ross, M Rowe, ICR Rowley, NW Schrader, DA Secomb, DL Serventy, TGD Shannon, RC Shearer, KG Simpson, PJ Slater, IJ Smales, DI Smedley, RJ Smith, JL Smith, KW Smith, GT Smith, CN Smithers, M Smyth, AK Smyth, PJ Spurge, AF Stewart, DA Stewart, A Stokes, RH Stranger, BRM Strong, PD Strong, H Sutherland, J Tagell, MK Tarburton, MRS Templeton, MT Templeton, T Templeton, RG Thoday, EB Thomas, DG Thomas, HF Thomas, GD Thomas, PJ Thomson, SC Tidemann, RA Tilt, A Tingay, WA Trudgen, LJ Turner, FWC Van Gessel, NW Vincent, WC Wakefield, E Wakefield, LE Wall, JE Walsh, RM Warneke, RWJ Warnock, MH Waterman Oam, DM Watson, I Watson, NF Weatherill, MS Webster, JA Welbergen, E Wheeler, JR Wheeler, A Whitehead, AC Williams, DJ Williams, L Willoughby, JT Willows, SJ Wilson, GB Winning, JCZ Woinarski, KA Wood, VJ Wood, R Woodell, SJ Wooller, NR Wright, E Wyndham, and RA Zann.

**Geographic Distributions**

Australian passerines were divided into two groups: sedentary (*n* = 292, taxa whose breeding range and year round range are largely the same with only local dispersal by juveniles) and definitive (*n* = 18, taxa that have defined breeding ranges that differ from their year round ranges) (^1^. For sedentary species we used all observations and for definitive species we limited observations to those during breeding (e.g. nest, eggs, young, or defined as breeding). We increased the spatial accuracy of breeding ranges by removing observations that were farther than 200 km from BirdLife’s species distribution polygons ^2^ (*n* = 279 species) and manually removing clearly erroneous observations that were well outside of known ranges (*n* = 15 species). To limit the duplication of observations at a given location, we reduced the spatial accuracy of records to 1 km, using Albers Equal Area Conic Projection, and kept only one observation of a species per 1 km x 1 km gridcell. This resulted in a total of 4,313,381 observations. The 310 passerine species were spread across 39 families, with the largest family, Meliphagidae (honeyeaters and chats), being represented by the most species (*n* = 73 species). The number of occurrences per species ranged from 7 to 144533, with a mean of 14037 and median of 3174.

To assess the effect of spatial resolution on observed species richness (number of species with breeding observations in a gridcell), we compared observed and estimated species richness in grid cells of 50 km x 50 km and 100 km x 100 km ^3^. Species richness was estimated using a bias-corrected Chao estimation ^4,5^ in R 3.3.2 ^6^ using the package *vegan 2.3-4* ^7^. We compared species richness by calculating a Completeness Index (CI): the ratio of the observed species richness to the estimated species richness. The 50 km analysis performed poorly when assessing all passerine species, with 12.7% of grid cells having CI < 0.7, while at the 100 km resolution only 6.2% of grid cells had CI < 0.7. Thus, all assessments of geographic patterns in this study are carried out for 100 km x 100 km grid cells. We excluded grid cells from our analysis when; (i) CI was less than 0.7, or (ii) there were fewer than 10 species with cup-shaped nests and 10 species with domed nests in a grid cell. After exclusions, a total of 713 grid cells were assessed. The number of grid cells occupied per species had a strong positive skew and ranged from 2 to 712 with a mean of 174 and median of 97. Species richness within 100 km x 100 km grid cells varied from 29 to 162 species, with a mean of 75 and a median of 69 (Fig. S1).


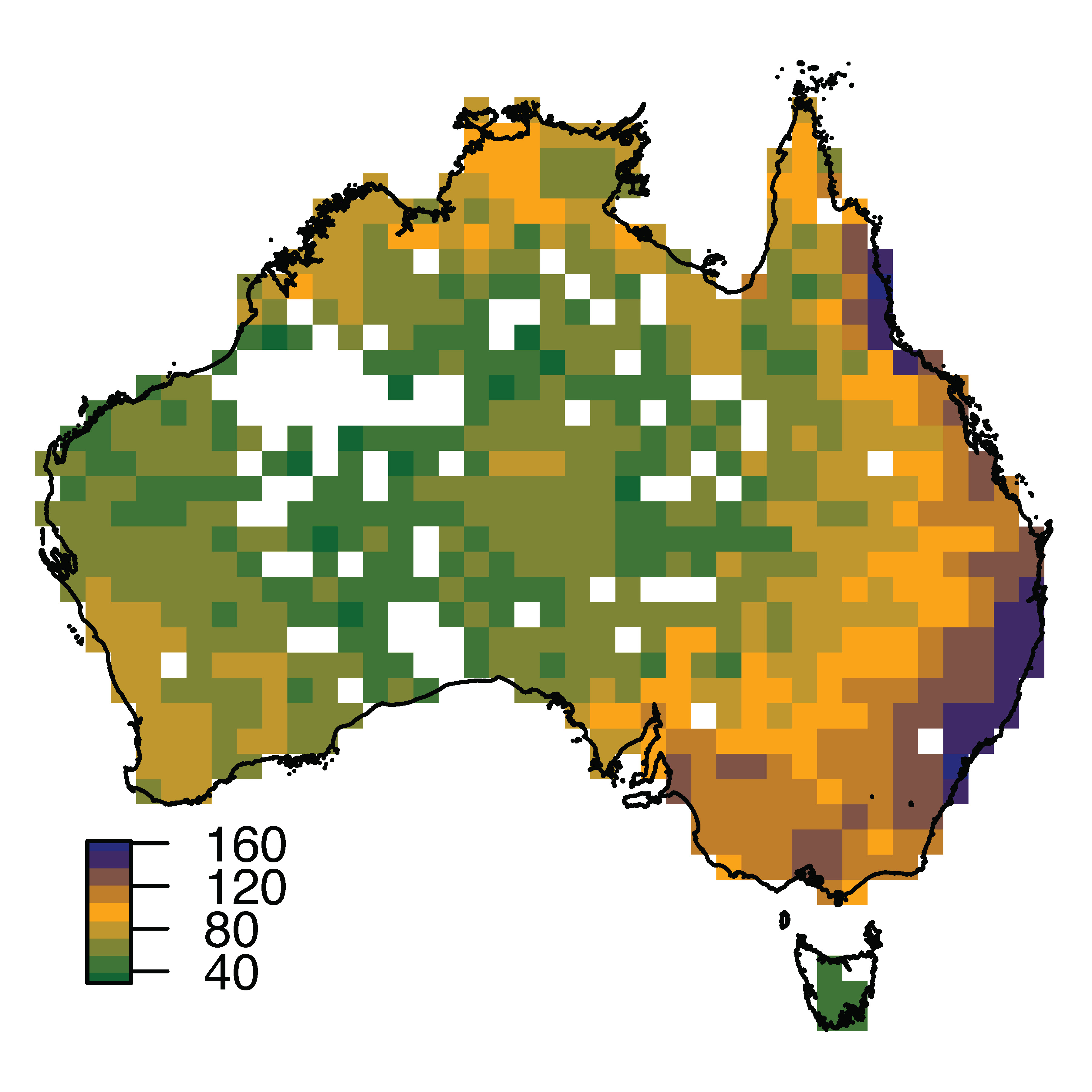


Figure S1. Variation in observed passerine species richness for breeding birds across the Australian continent. Observed species richness is for 100 km x 100 km grid cells. White grid cells either have less than ten species in domed or cup-shaped nests or do not meet the required Completeness Index (i.e. the ratio of the observed species richness to the estimated species richness). Maps were created in R 3.3.2 ^6^ using raster ^8^ and base plotting function. Digital boundaries of Australia are from Australian Standard Geographical Classification (ASGC) (cat. no. 1216.0).

**Variation in egg elongation using a mixed-effect model**

Nest type explained 4% of the variation in egg elongation across all species ([F (2, 95.6) = 3.7, p < 0.05]). Cavity-nesting species had significantly less elongated eggs (1.32 ± 0.02, *n* = 16) than species that nest in cup-shaped and domed nests (1.37 ± 0.02, *n* = 191; 1.37 ± 0.02, *n* = 101, respectively. There was no significant difference between cup-shaped and dome species.

**References**

1. Garnett, S. T. *et al.* Biological, ecological, conservation and legal information for all species and subspecies of Australian bird. *Sci. Data* **2:150061,** (2015).

2. BirdLife International and NatureServe. *Bird species distribution maps of the world*. (BirdLife International, Cambridge, UK and NatureServe, Arlington, USA, 2015).

3. Soberón, J., Jiménez, R., Golubov, J. & Koleff, P. Assessing completeness of biodiversity databases at different spatial scales. *Ecography* **30,** 152–160 (2007).

4. Chao, A. Estimating the Population Size for Capture-Recapture Data with Unequal Catchability. *Biometrics* **43,** 783–791 (1987).

5. Chiu, C.-H., Wang, Y.-T., Walther, B. A. & Chao, A. An improved nonparametric lower bound of species richness via a modified good–turing frequency formula. *Biometrics* **70,** 671–682 (2014).

6. R Core Team. *R: A Language and Environment for Statistical Computing. R Foundation for Statistical Computing. https://www.R-project.org/*. (2016).

7. Oksanen, J. *et al.* vegan: Community Ecology Package, R package version 2.3-4. (2016).

8. Hijmans, R. J. raster: Geographic Data Analysis and Modeling. R package version 2.5-8. (2016).
